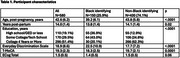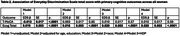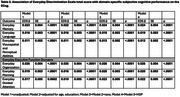# Discrimination, hypertensive disorders of pregnancy, and cognition in midlife women

**DOI:** 10.1002/alz70860_107463

**Published:** 2025-12-23

**Authors:** C. Elizabeth Shaaban, Ashley V Hill, Alyssa Smith, Samantha Bryan, Robin Gandley, Elizabeth Swart, Janet M Catov

**Affiliations:** ^1^ University of Pittsburgh, Pittsburgh, PA, USA; ^2^ University of Pittsburgh Alzheimer's Disease Research Center (ADRC), Pittsburgh, PA, USA; ^3^ University of Illinois Chicago, Chicago, IL, USA; ^4^ Magee‐Womens Research Institute and Foundation, Pittsburgh, PA, USA

## Abstract

**Background:**

Profound and persistent disparities in late‐life brain health exist by gender/sex and racialized group. Hypertensive disorders of pregnancy (HDP), which disproportionately impact Black women, have recently been recognized as a sex‐specific midlife risk factor for Alzheimer's disease and related dementias (ADRD). Furthermore, discrimination is an emerging risk factor for both HDP and later poor cognitive health. We aimed to assess associations of discrimination with cognition in midlife women, independent of HDP.

**Method:**

Participants (*n* = 580) were recruited from a clinical cohort at Magee‐Womens Hospital, Pittsburgh, PA. Births occurred 2008‐2012, with demographics, the Everyday Discrimination Scale (EDS; a measure of day‐to‐day discrimination experiences), the telephone Montreal Cognitive Assessment (T‐MoCA; an objective cognitive measure), and the Everyday Cognition Scale (ECog; a subjective cognitive measure) completed in 2024. We tested association of discrimination with cognition using linear regression models adjusting for age, education, race, and HDP. We explored associations of discrimination with domains of subjective cognitive performance on the ECog including: organization, planning, divided attention, memory, language, and visuospatial and perceptual abilities.

**Result:**

Participants were 42.6±6.2 years old, 14.0±1.4 years post‐partum, and 51.4% had≥college education (Table 1). Self‐identified race was Black (*n* = 150 (25.9%)) compared to non‐Black participants [*n* = 430 (74.1%), White (*n* = 415), Latina (*n* = 5), Asian (*n* = 9), or American Indian (*n* = 1)] due to small numbers of some groups. Black women more commonly endorsed race as the cause of the discrimination vs. non‐Black women (62.7% vs. 5.6%, *p* < .0001), while non‐Black women more commonly endorsed gender as the cause of the discrimination vs. Black women (32.6% vs. 18.0%, *p* = 0.007). In fully adjusted models, EDS was not significantly associated with T‐MoCA score (*p* = 0.59, Table 2), but greater discrimination reported on the EDS was significantly associated with greater (worse) ECog scores (*p* < .0001). Greater EDS was significantly associated with worse ECog scores across all domains examined (Table 3). In fully adjusted models, the largest effect sizes were seen in everyday organization, memory, divided attention, and planning.

**Conclusion:**

Subjective cognitive measures (vs. objective) were more sensitive to associations with discrimination. Executive and memory domains were most strongly associated, indicating that discrimination may have implications for day‐to‐day function and risk of cognitive impairment before late‐life.